# Reversible protein aggregation as cytoprotective mechanism against heat stress

**DOI:** 10.1007/s00294-021-01191-2

**Published:** 2021-06-06

**Authors:** Paola Gallardo, Silvia Salas-Pino, Rafael R. Daga

**Affiliations:** 1 Centro Andaluz de Biología del Desarrollo, Universidad Pablo de Olavide-Consejo Superior de Investigaciones Científicas, Junta de Andalucía, Seville, Spain; 2 European Research Institute for the Biology of Ageing, University of Groningen, University Medical Center Groningen, Groningen, Netherlands

## Abstract

Temperature fluctuation is one of the most frequent threats to which organisms are exposed in nature. The activation of gene expression programs that trigger the transcription of heat stress-protective genes is the main cellular response to resist high temperatures. In addition, reversible accumulation and compartmentalization of thermosensitive proteins in high-order molecular assemblies are emerging as critical mechanisms to ensure cellular protection upon heat stress. Here, we summarize representative examples of membrane-less intracellular bodies formed upon heat stress in yeasts and human cells and highlight how protein aggregation can be turned into a cytoprotective mechanism.

## Introduction

High temperature induces the unfolding and exposure of hydrophobic stretches in thermosensitive proteins, which can establish non-native intra- and inter-molecular interactions leading to aggregation into high-order protein assemblies (Chiti and Dobson 2006; Kammerer et al. 2004; Tyedmers et al. 2010). Furthermore, a significant part of the proteome contains intrinsically disordered domains (IDD) (Oldfield and Dunker 2014; Uversky 2017). IDDs usually show high structural flexibility and, under environmental perturbations, such as high temperature, they can acquire new folding states that make them more prone to establish interactions with other proteins. This leads to phase separation and the concentration of molecules in intracellular condensates (Alberti and Hyman 2021; Dyson and Wright 2005; Fomicheva and Ross 2021; Franzmann and Alberti 2019; Uversky and Dunker 2010). The dynamic properties of IDDs also contribute to the formation of protein aggregates under more extreme stress conditions (Kim et al. 2013; Molliex et al. 2015; Patel et al. 2015).

Cells respond to protein folding stress by activating the heat stress response (HSR), a transcriptional program that induces the expression of heat stress-response proteins (HSPs) such as chaperones and other cytoprotective factors, which boost the refolding of damaged proteins or their clearance by proteolytic mechanisms (Richter et al. 2010; Verghese et al. 2012). However, conditions of high proteotoxic stress that overcome the protein quality-control system may result in persistent protein aggregates which have been classically linked to a pervasive cellular decline and the development of degenerative and age-related diseases (pathological aggregation) (Diaz-Villanueva et al. 2015; Hipp et al. 2014; Klaips et al. 2018; Schneider and Bertolotti 2015). Interestingly, the accumulation of proteins in aggregates has also been more recently described as an organized and reversible process that displays cytoprotective functions (physiological aggregation) (Audas et al. 2016; Franzmann and Alberti 2019; Marijan et al. 2019; Tyedmers et al. 2010). This protective protein aggregation results in the formation of a variety of specific membrane-less inclusion bodies or biomolecular condensates, with different cellular locations, physico-chemical properties, and functions (Gallardo et al.^2020^; Shav-Tal et al. 2005; Sontag et al. 2017; Tyedmers et al. 2010; van Leeuwen and Rabouille 2019; Wallace et al. 2015; Wang et al. 2018). This review summarizes current knowledge on biomolecular condensates induced by HS in yeasts and humans with a focus on the recently described nucleolar rings (NuRs) in *Schizosaccharomyces pombe.* We discuss the function of the Hsf1-dependent HSR in the regulation of NuRs and its role in the maintenance of cell viability in high proteotoxic conditions such as acute HS.

## Stress granules and cytoplasmic protein aggregation centers.

**Fig. 1:**
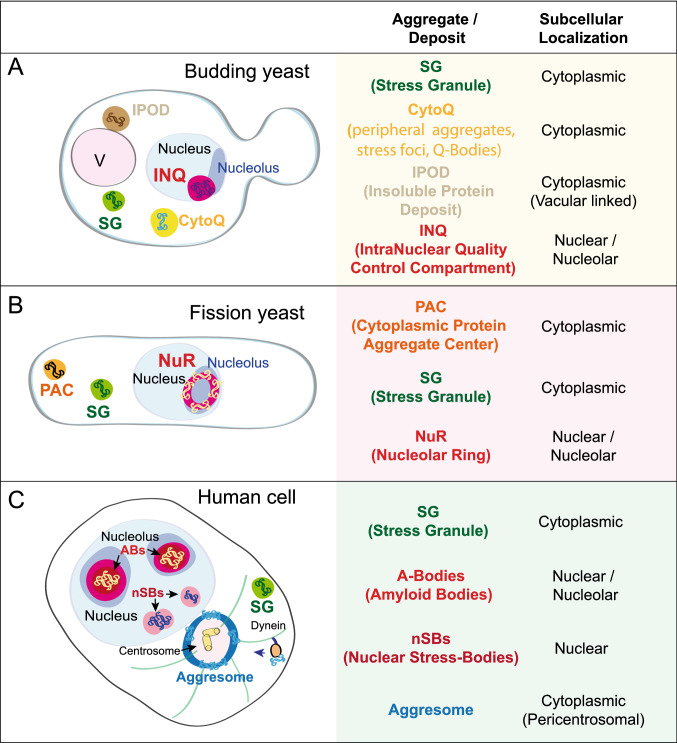
Cytoprotective aggregation centers under HS. Schematic representation of different intracellular biomolecular condensates formed in *Saccharomyces cerevisiae*, *Schizosaccharomyces pombe*, and human cells at different cellular locations upon HS. **A** Biomolecular condensates in *S. cerevisiae* upon HS. SGs and CytoQ encompass different cytoplasmic condensates, which contain non-terminally misfolded proteins mainly destined for refolding after the stress, similar to the INQ formed in the nucleus adjacent to the nucleolus. IPODs, however, are aggregate deposits formed in the cytoplasm adjacent to the vacuole, which accumulate terminally misfolded proteins destined for degradation. **B** Two types of cytoplasmic condensates have been described in *S. pombe;* PACs and SGs. PACs form upon mild HS and accumulate non-terminally misfolded proteins whose fate is refolding. PACs have been proposed to constitute the precursors of SGs formed upon more severe stress conditions. In the nucleus, misfolded proteins accumulate at NuRs upon acute HS. NuRs form at the nucleolar region and function as reversible protein sequestering centers. Upon stress relief, NuRs dissolution is linked to the restoration of cell growth. **C** Upon stress conditions, human cells form different cytoplasmic deposition sites, such as SGs or aggresomes. Aggresomes represent sites of terminal aggregation of damaged proteins whose destiny is degradation by autophagic mechanisms. In the nucleus, human cells protect thermosensitive proteins by their coalescence along with lncRNAs transcribed from ribosomal intergenic spacers in nucleolar structures known as amyloid bodies (ABs). AB are reversible structures and have been shown to promote local nuclear translation during the stress. On the other hand, nSBs are nuclear assemblies of RNA and proteins formed upon HS which function by sequestering transcription-related factors facilitating the HS-induced inhibition of bulk RNA transcription

One of the most conserved and well-described inclusion bodies are the cytoplasmic stress granules (SGs) that are rapidly formed as a response to several environmental stresses, including high temperature (Fig. 1) (Buchan and Parker 2009). SGs are biomolecular condensates containing mainly RNA-binding proteins (RBPs), translationally repressed mRNAs, translation factors, and 40S ribosomal subunits. SG formation is driven by the unfolding and promiscuous interactions of IDDs present in RNA-binding proteins (RBPs) and involves a number of post-translational protein modifications (Hofmann et al. 2021; Molliex et al. 2015). SGs are dynamic structures that contribute to the repression of protein synthesis upon stress and are considered sites of mRNA triage, sorting towards the decay or the storage of mRNAs (Alberti et al. 2017; Anderson and Kedersha 2009).

Other cytosolic biomolecular condensates termed protein aggregate centers (PACs) have been recently described to form in the fission yeast upon exposure to a mild HS (37 ℃) (Fig. 1B) (Cabrera et al. 2020). PACs are dynamic assemblies that contain chaperones and components of the translational machinery; however, misfolded proteins also accumulate at PACs and this is required to avoid their degradation during the HS. Therefore, PACs have been proposed to protect these non-terminally misfolded proteins from degradation. PACs behave as liquid-like condensates that change to a more compacted state upon incubation of cells at higher temperatures, which suggests that PACs can function as seeds for SG formation. The assembly and disassembly of SGs and PACs are regulated by Hsp70 chaperones (Boronat et al. 2021; Cabrera et al. 2020; Cherkasov et al. 2013; Kroschwald et al. 2015; Mateju et al. 2017; O'Driscoll et al. 2015; Walters et al., 2015), and the disaggregase Hsp104 is key for their dissolution (Cabrera et al. 2020; Cherkasov et al. 2013; Kroschwald et al. 2015).

## Insoluble protein deposit, CytoQ, and Intranuclear quality-control compartment.

Other protein deposition centers have been observed in *Saccharomyces cerevisiae* as a result of HS. These include insoluble protein deposit (IPOD) and CytoQ in the cytoplasm, and intra-nuclear quality-control compartment (INQ) in the nucleus (Fig. 1A). IPODs are perivacuolar deposits constituted by immobile, terminally aggregated proteins, including the amyloidogenic prion proteins Rnq1 and Sup35 (Kaganovich et al. 2008; Kumar et al. 2016), while CytoQ refers to different cytoplasmic condensates containing cytosolic non-terminally misfolded proteins. On the contrary, INQ, also termed juxtanuclear quality-control compartment (JUNQ), localize inside the nucleus, adjacent to the nucleolus, and harbor nuclear and cytosolic misfolded proteins (Kaganovich et al. 2008; Miller et al. 2015). CytoQ and INQ share similarities in their formation, which is dependent on cell-compartment-specific aggregases: the cytoplasmic small HSP (sHSP) Hsp42 for CytoQs and the nuclear aggregase Btn2 for INQs. Hsp42 and Btn2 function as sorting factors that promote the partitioning of misfolded proteins into CytoQs or INQs, respectively (Malinovska et al. 2012; Miller et al. 2015; Specht et al. 2011), and their activities are key to avoid the overload of the proteostatic capacity and, consequently, to prevent the loss of cell viability. Non-terminally unfolded proteins stored at CytoQs and INQs will be mainly targeted to the refolding pathway (Ho et al. 2019; Miller et al. 2015; Wallace et al. 2015), whereas terminally misfolded substrates accumulated at IPODs are either targeted for clearance (mainly by autophagy) or diluted by cell division (Kaganovich et al. 2008). Of note, in both cases, the Hsp70 chaperone system and the disaggregase Hsp104 participate in their sorting and re-solubilization (Gallina et al. 2015; Ho et al. 2019; Kaganovich et al. 2008; Malinovska et al. 2012; Miller et al. 2015; O'Driscoll et al. 2015).

## Aggresomes, nuclear stress bodies, and amyloid bodies

Human cells have also been shown to form specialized inclusion bodies for sequestering misfolded proteins under HS. These include SGs and aggresomes in the cytoplasm, and nuclear stress bodies (nSBs) and amyloid bodies (AB) in the nucleus (Fig. 1C).

Aggresome assembly depends on the active delivery (by dynein motor complex) and the accumulation of aggresomal particles containing misfolded proteins near the centrosome (Johnston et al. 1998) (Fig. 1). Aggresomes recruit chaperones, ubiquitination enzymes, and proteasomes and, in conditions of insufficient proteasome degradation, the prolonged presence of aggresomes results in their autophagic clearance (Johnston et al. 1998; Tyedmers et al. 2010). Therefore, they are considered as garbage depositories that aid in the clearance of terminally aggregated proteins (Kawaguchi et al. 2003; Tyedmers et al. 2010).

On the other hand, ABs are formed in the nucleus by the rapid and reversible interaction between heterogeneous proteins and ribosomal intergenic non-coding RNA (rIGSRNA). rIGSRNAs are expressed in a stress-inducible manner and act as the seeding elements for AB formation in subnuclear foci, trapping, and immobilizing proteins that are characterized by their insolubility. Upon stress relief, ABs are disaggregated in an HSP70-dependent manner and their components return to their normal localization (Audas et al. 2016, 2012). Interestingly, a new and unexpected function has been recently ascribed to ABs as solid-like condensates that support nuclear translation of Hsf1 targets during acidosis and HS (Theodoridis et al. 2021).

Another subnuclear foci that is found exclusively in primates upon heat and chemical stresses are the nuclear stress bodies (nSBs) (Biamonti and Vourc'h 2010). nSB formation is initiated by binding of Hsf1 to pericentric tandem repeats of satellite III (Sat 3). Hsf1-dependent transcription of Sat 3 transcripts promotes the binding and sequestration of transcription factors and RBPs to these long non-coding RNAs (lncRNAs). Sat3 transcripts tend to stay associated with the transcription site, forming subnuclear foci that concentrate and sequester several nuclear factors required for transcription, mainly at the 9q12 locus (Biamonti and Vourc'h 2010). nSBs do not seem to colocalize with Hsf1 canonical targets of the HSR, suggesting that nSB formation is not required for the transcription of HSR genes (Jolly et al. 1997). However, nSB assembly is required to sustain the HS-induced transcriptional repression and to maintain cell viability after the HS (Goenka et al. 2016) and, therefore, they have been proposed to function as sequestering centers which facilitate the downregulation of general transcription during the stress.

## Nucleolar rings

In fission yeast, acute heat stress (42 ℃), which prevents bulk protein synthesis and blocks general mRNA metabolism (Cabrera et al. 2020; Gallardo et al. 2020; Ribeiro et al. 1997), results in a dramatic reorganization of the nucleus. These nuclear architectural changes include nucleolar contraction and the formation of ring-shaped nucleolar aggregation centers, named Nucleolar Rings (NuR) (Gallardo et al. 2020). NuRs assemblies contain a wide and functionally divergent group of nuclear factors, including factors involved in mRNA processing and export, nuclear pore complex (NPC) components, mRNA, chromatin-associated factors, and cell cycle regulators. The formation of these assemblies is triggered by HS-induced unfolding and aggregation of proteins and RNAs (Gallardo et al. 2020). While the aggregation of cell cycle factors at NuRs could contribute to the arrest of cell division observed under this extreme temperature, the rearrangement of the NPC and mRNA machinery could be responsible for the block of housekeeping mRNA metabolism and export and for the formation of a reservoir of messenger ribonucleoprotein (mRNPs) particles, which would be ready for export once stress conditions cease. This rearrangement bears similarities with the compositional changes of mRNPs under heat stress described for other organisms, including budding yeast and higher eukaryotes (Bond 1988; Bracken and Bond 1999; Hochberg-Laufer et al. 2019; Kay et al. 1987; Lutz et al. 1988; Mahl et al. 1989; Mayrand and Pederson, 1983; Sadis et al. 1988). In fact, in *S. cerevisiae*, heat stress leads to the uncoupling of several RBPs from the mRNA and their accumulation in nuclear foci, in a manner dependent on the nucleoporins Mlp1 and Mlp2. This reorganization has been proposed to prevent the quality control and export of regular mRNAs, while promoting the export of HS mRNAs (Carmody et al. 2010; Zander et al. 2016; Zander and Krebber 2017).

The study of NuRs has shed light on the dynamic nature of these stress-induced assemblies. NuRs are formed by the rapid aggregation of multiple nuclear proteins, which remain mostly immobile, while the stress persists. However, when the stress is relieved, NuRs disaggregate and their multiple components relocate back to their functional localization (Gallardo et al. 2020). This shows that fission yeast cells can modulate protein aggregation, turning deleterious protein aggregates into cytoprotective protein sequestering centers which contribute to maintain cellular homeostasis.

Hsf1 is the main transcription factor involved in the activation of the HSR (Akerfelt et al. 2010). Upon incubation of fission yeast cells at 42 ℃, Hsf1 rapidly accumulates in the nucleoplasm; however, it does not localize at NuRs. Interestingly, once the heat stress is over, cell growth recovery tightly correlates with a peak in Hsf1 expression (Gallardo et al. 2020). This delayed Hsf1 upregulation is also observed in *S. cerevisiae* during the recovery from severe heat shock (Yamamoto et al. 2008). Furthermore, a partial Hsf1 depletion significantly delays NuRs dissolution and cell growth reinitiation during the recovery from acute HS. This suggests that Hsf1 expression during the acute HS is limited, and that a burst in newly synthesized Hsf1 during the recovery period is required to trigger the refolding of most heat-denatured proteins in order to reactivate cellular metabolism (Gallardo et al. 2020; Yamamoto et al. 2008).

Hsf1 activation leads to the expression of HSPs, such as chaperones and disaggregases (Akerfelt et al. 2010). These proteins localize at aggregates and are responsible for aggregate dissolution once the stress is over. In addition, chaperones are commanders in the triage of aggregated proteins for recycling or degradation during the recovery from HS (Balchin et al. 2016; Escusa-Toret et al. 2013; Hartl et al. 2011; Malinovska et al. 2012; Mogk et al. 2015; Vabulas et al. 2010). Consistently, NuRs accumulate several HSPs, such as the Hsp70 homologs Ssa1 and Ssa2, and the disaggregase Hsp104.

Importantly, Hsp104 is required for NuR aggregate dissolution, for the relocalization of NuRs components, and for the resumption of cell growth after acute HS (Gallardo et al. 2020). This is in agreement with previous studies that have demonstrated that Hsp104, in concert with the chaperones Hsp70/Hsp40, functions in the reassembly of small nuclear RNPs after HS (Bracken and Bond 1999; Cherkasov et al. 2013; Haslbeck et al. 2005). Interestingly, a recent study in budding yeast shows that the Hsp70/Hsp40 chaperone partners aid in the solubilization of intra-nuclear inclusions formed after incubation of the cells at 42 ℃, independently on Hsp104. In these conditions, Hsp40 and Hsp104 compete for protein disaggregation, targeting proteins towards turnover or refolding, respectively (den Brave et al. 2020). Metazoans lack Hsp104 orthologues and the disaggregase activity is accomplished by Hsp70 and Hsp110, among others (Mogk et al. 2015; Rampelt et al. 2012; Shorter 2011).

In conclusion, fission yeast NuRs are reversible molecular assemblies rapidly formed upon acute heat stress by the aggregation of nuclear and nucleolar factors along with RNA. Their formation correlates with nucleolar contraction and the inhibition of cell growth and their Hsp104-dependent dissolution is required for cell growth restoration once the heat stress is relieved. Therefore, NuRs can be considered emergency storage deposits for thermolabile proteins and its assembly might contribute to the inhibition of nuclear functions under acute HS.

## Concluding remarks

As discussed above, HS elicits the formation of a variety of high-order-molecular assemblies, which are formed both in the cytoplasm and the nucleus, by the promiscuous interaction of misfolded proteins, IDD-containing proteins and usually RNA. Although the functions of all these intracellular protein deposits are still under investigation, they share a common role as protective mechanisms preserving cellular homeostasis during stress conditions and promoting cell survival. The concentration of proteins in biomolecular condensates contributes to inactivate unneeded or energetically consuming cellular functions and avoids the interference of misfolded proteins with stress-response cellular pathways. The local recruitment and concentration of HSPs at these condensates facilitates the refolding or clearance of stress-sensitive proteins and can also promote the efficiency of essential processes, such as translation in the case of ABs.

Understanding how chaperones modulate and reverse protein aggregation is of prime importance, since the formation of amyloid deposits has been linked to the development of a variety of degenerative diseases, such as Amyotrophic Lateral Sclerosis, Alzheimer's, Huntington’s, or Parkinson's disease, among others (Chiti and Dobson 2017). In addition, recent advances have shown that aging is associated with progressive exhaustion of the proteostatic capacity of cells and the formation of pathogenic aggresomes, which compromises multiple cellular processes (Hipp et al. 2019, 2014; Moreno-Blas et al. 2018; Morimoto 2008; Thiruvalluvan et al. 2020). Therefore, achieving a better knowledge of what factors and mechanisms are involved in the aggregation and disaggregation processes would be extremely valuable for future therapeutic strategies of age-related diseases. In this sense, yeasts as model organisms represent a valuable tool for genetic or drug screening purposes to find new modulators that could potentially alleviate pathologic aggregation in humans.
